# Supplementary material for: The role of minority language bilingualism in spotting agreement attraction errors: Evidence from Italian varieties
Source: PLoS One. 2024 Feb 27;19(2):e0298648. doi: 10.1371/journal.pone.0298648 (PMC10898745; doi:10.1371/journal.pone.0298648)
Supplement: S1 Table — Accuracy rates are set as the dependent variable, language groups (i.e., “monolingual”, “bilingual”, “Agrigentino”, and “Pavese”) are set as fixed factors, while animacy, register, gender, and age are set as control factors. (PDF) [file pone.0298648.s001.pdf]

Effect	Estimate	SE	z	p	by-participant SD	by-item SD
(Intercept)	1.636388	0.228756	7.15343	8.463602	1.9459	0.5822
Comparison between monolingual and Agrigentino groups	-1.18852	0.342797	-3.46712*	0.000526		
Comparison between monolingual and Pavese groups	0.724389	0.38912	1.861606	0.062659		
Comparison between monolingual and bilingual groups	0.324106	0.344428	0.941	0.346705		
Animacy	-0.1721	0.102849	-1.67329	0.09427		
Register	-0.21219	0.102904	-2.06203*	0.039205		
Gender	-0.20616	0.212744	-0.96907	0.332511		
Age	-0.70609	0.23108	-3.05561*	0.002246		

S1 Table. Fixed and random effects from the GLME of Accuracy, with the monolingual group as the baseline. Accuracy rates are set as the dependent variable, language groups (i.e., “monolingual”, “bilingual”, “Agrigentino”, and “Pavese”) are set as fixed factors, while animacy, register, gender, and age are set as control factors.
