# Supplementary material for: The role of minority language bilingualism in spotting agreement attraction errors: Evidence from Italian varieties
Source: PLoS One. 2024 Feb 27;19(2):e0298648. doi: 10.1371/journal.pone.0298648 (PMC10898745; doi:10.1371/journal.pone.0298648)
Supplement: S2 Table — Accuracy rates are set as the dependent variable, language groups (i.e., “monolingual”, “bilingual”, “Agrigentino”, “Pavese”) are set as fixed factors, while animacy, register, gender, and age were set as control factors. (PDF) [file pone.0298648.s002.pdf]

Effect	Estimate	SE	z	p	by-participant SD	by-item SD
(Intercept)	1.636381	0.228752	7.153502	8.459132	1.9459	0.5821
Comparison between Pavese and monolingual groups	0.140137	0.382405	0.366463	0.71402		
Comparison between Pavese and Agrigentino groups	-1.18853	0.342773	-3.46739	0.000526*		
Comparison between Pavese and bilingual group	0.324044	0.344425	0.940825	0.346794		
Animacy	-0.17212	0.102846	-1.6736	0.09421		
Register	-0.2122	0.102902	-2.06217	0.039191*		
Gender	-0.20613	0.21274	-0.96891	0.33259		
Age	-0.70602	0.23107	-3.05544	0.002247*		

S2 Table. Fixed and random effects from the second GLME of Accuracy, with the Italian-Pavese bidialectal group set as the baseline. Accuracy rates are set as the dependent variable, language groups (i.e., “monolingual”, “bilingual”, “Agrigentino”, “Pavese”) are set as fixed factors, while animacy, register, gender, and age were set as control factors.
