# Supplementary material for: The role of minority language bilingualism in spotting agreement attraction errors: Evidence from Italian varieties
Source: PLoS One. 2024 Feb 27;19(2):e0298648. doi: 10.1371/journal.pone.0298648 (PMC10898745; doi:10.1371/journal.pone.0298648)
Supplement: S3 Table — (PDF) [file pone.0298648.s003.pdf]

Factor	GVIF	Df	GVIF^(1/(2*Df))
Group	1.441456	3	1.062837536
Animacy	1.000054	1	1.000026796
Register	1.000065	1	1.00003262
Gender	1.056288	1	1.027758777
Age	1.399619	1	1.183055012

S3 Table. VIF for the first GLME of Accuracy (S1 Table), with the monolingual group as the baseline.
