# Supplementary material for: The role of minority language bilingualism in spotting agreement attraction errors: Evidence from Italian varieties
Source: PLoS One. 2024 Feb 27;19(2):e0298648. doi: 10.1371/journal.pone.0298648 (PMC10898745; doi:10.1371/journal.pone.0298648)
Supplement: S4 Table — (PDF) [file pone.0298648.s004.pdf]

Factor	GVIF	Df	GVIF^(1/(2*Df))
Group	1.44141	3	1.062831885
Animacy	1.000054	1	1.000026801
Register	1.000065	1	1.000032662
Gender	1.056281	1	1.027755472
Age	1.399575	1	1.183036283

S4 Table. VIF for the first GLME of Accuracy (S2 Table), with the Italian-Pavese bidialectal group set as the baseline.
