# Supplementary material for: The role of minority language bilingualism in spotting agreement attraction errors: Evidence from Italian varieties
Source: PLoS One. 2024 Feb 27;19(2):e0298648. doi: 10.1371/journal.pone.0298648 (PMC10898745; doi:10.1371/journal.pone.0298648)
Supplement: S5 Table — Log-transformed RTs are set as the dependent variable, language groups (i.e., “monolingual”, “bilingual”, “Agrigentino”, “Pavese”) and “Judgement” are set as fixed factors. Animacy, register, gender, and age are set as control factors. (PDF) [file pone.0298648.s005.pdf]

Effect	Estimate	SE	t	p	by-participant SD	by-item SD
Intercept	3.17022	0.018932	167.4537	1.104342	0.17394	0.03158
Comparison between monolingual and Agrigentino groups	0.054767	0.030264	1.809666	0.073268		
Comparison between monolingual and Pavese groups	-0.07092	0.033487	-2.11772*	0.036623		
Comparison between monolingual and bilingual groups	-0.02693	0.030185	-0.89227	0.374345		
Judgement	0.053879	0.005734	9.397239*	< 0.000		
Animacy	0.000162	0.006459	0.025051	0.980149		
Register	-0.00039	0.006463	-0.06014	0.952368		
Gender	0.027323	0.01864	1.46577	0.145787		
Age	0.076381	0.020261	3.76992*	0.000273		
Judgement * Comparison between monolingual and Agrigentino groups	-0.01951	0.008653	-2.25437*	0.024225		
Judgement * Comparison between monolingual and Pavese groups	-0.00535	0.010848	-0.49279	0.622191		
Judgement * Comparison between monolingual and bilingual groups	-0.0105	0.009469	-1.10897	0.267506		

S5 Table. Fixed and random effects from the LME of log-transformed RTs, with the monolingual group as the baseline. Log-transformed RTs are set as the dependent variable, language groups (i.e., "monolingual", "bilingual", "Agrigentino", "Pavese") and "Judgement" are set as fixed factors. Animacy, register, gender, and age are set as control factors.
