# Supplementary material for: The role of minority language bilingualism in spotting agreement attraction errors: Evidence from Italian varieties
Source: PLoS One. 2024 Feb 27;19(2):e0298648. doi: 10.1371/journal.pone.0298648 (PMC10898745; doi:10.1371/journal.pone.0298648)
Supplement: S6 Table — Log-transformed RTs are set as the dependent variable, language groups (i.e., “monolingual”, “bilingual”, “Pavese”, “Agrigentino”) and “Judgement” are set as fixed factors. Animacy, register, gender, and age are set as control factors. (PDF) [file pone.0298648.s006.pdf]

Effect	Estimate	SE	t	p	by-participant SD	by-item SD
Intercept	3.169806	0.018933	167.4251	1.131487	0.17394	0.03158
Comparison between Agrigentino and monolingual groups in RTs	0.042721	0.033343	1.281236	0.203		
Comparison between Agrigentino and Pavese groups in RTs	-0.07086	0.033485	-2.11619*	0.036757		
Comparison between Agrigentino and bilingual groups in RTs	-0.02683	0.030183	-0.88877	0.376215		
Judgement	0.053714	0.005716	9.397239*	< 0.000		
Animacy	0.000162	0.006459	0.025051	0.980149		
Register	-0.00039	0.006463	-0.06014	0.952368		
Gender	0.027323	0.01864	1.46577	0.145787		
Age	0.07642	0.020271	3.76992*	0.000273		
Judgement * Comparison between Agrigentino and monolingual groups	0.035246	0.009932	3.548694*	0.000391		
Judgement * Comparison between Agrigentino and Pavese groups	-0.00533	0.010815	-0.49279	0.622191		
Judgement * Comparison between Agrigentino and bilingual groups	-0.01047	0.00944	-1.10897	0.267506		

S6 Table. Fixed and random effects from the first LME of log-transformed RTs, with the Italian-Agrigentino bidialectal group set as the baseline. Log-transformed RTs are set as the dependent variable, language groups (i.e., “monolingual”, “bilingual”, “Pavese”, “Agrigentino”) and “Judgement” are set as fixed factors. Animacy, register, gender, and age are set as control factors.
