# Supplementary material for: The role of minority language bilingualism in spotting agreement attraction errors: Evidence from Italian varieties
Source: PLoS One. 2024 Feb 27;19(2):e0298648. doi: 10.1371/journal.pone.0298648 (PMC10898745; doi:10.1371/journal.pone.0298648)
Supplement: S8 Table — (PDF) [file pone.0298648.s008.pdf]

Factor	GVIF	Df	GVIF^(1/(2*Df))
Group	1.427898	3	1.061164902
Judgement	1.075887	1	1.037249714
Animacy	1.001349	1	1.000674323
Register	1.002767	1	1.001382758
Gender	1.054148	1	1.026717018
Age	1.382452	1	1.175777187
Group x Judgement	1.071595	3	1.011591389

S8 Table. VIF for the first LME of log-transformed RTs (S6 Table), with the Italian-Agrigentino bidialectal group as the baseline.
