# Supplementary material for: The role of minority language bilingualism in spotting agreement attraction errors: Evidence from Italian varieties
Source: PLoS One. 2024 Feb 27;19(2):e0298648. doi: 10.1371/journal.pone.0298648 (PMC10898745; doi:10.1371/journal.pone.0298648)
Supplement: S9 Table — Accuracy rates are set as the dependent variable. Language group (i.e., “Agrigentino”, “Pavese”, “bilingual”), “% of use of Italian”, “% of use of the L2”, and “% of switching” are set as fixed factors in the model and their interactions are also reported. Animacy, register, gender, and age are set as control factors. (PDF) [file pone.0298648.s009.pdf]

Effect	Estimate	SE	z	p	By-participant SD	By-item SD
Intercept	1.830894	0.449259	4.075368	0.000045	2.0262	0.5809
Comparison between bilingual and Agrigentino groups	-1.02866	0.524128	-1.96261	0.049692*		
Comparison between bilingual and Pavese groups	0.281209	0.584696	0.48095	0.630552		
% of language switching	0.033485	0.322411	0.103857	0.917282		
% Italian language use	0.276591	0.322234	0.858355	0.390696		
% L2 language use	-0.64167	0.474529	-1.35223	0.176303		
Animacy	-0.15334	0.105937	-1.44751	0.147754		
Register	-0.2398	0.10606	-2.26101	0.023759*		
Gender	-0.37387	0.299119	-1.24989	0.21134		
Age	-0.69412	0.263821	-2.63101	0.008513*		
% of switching - Comparison between bilingual and Agrigentino groups	0.028767	0.434299	0.066237	0.947189		
% of switching - Comparison between bilingual and Pavese groups	0.433908	0.486839	0.891276	0.372781		
% of use of Italian - Comparison between bilingual and Agrigentino groups	-0.24568	0.409015	-0.60067	0.548058		
% of use of Italian - Comparison between bilingual and Pavese groups	-0.32754	0.482162	-0.67931	0.496943		
% of use of L2 - Comparison between bilingual and Agrigentino groups	-0.7593	0.649724	-1.16865	0.242545		

% of use of L2 - Comparison between bilingual and Pavese groups	-0.25666	0.734922	-0.34924	0.726913

S9 Table. Fixed and random effects from the second GLME of Accuracy, with the Italian-Pavese bidialectal group as the baseline. Accuracy rates are set as the dependent variable. Language group (i.e., “Agrigentino”, “Pavese”, “bilingual”), “% of use of Italian”, “% of use of the L2”, and “% of switching” are set as fixed factors in the model and their interactions are also reported. Animacy, register, gender, and age are set as control factors.
