# Supplementary material for: The role of minority language bilingualism in spotting agreement attraction errors: Evidence from Italian varieties
Source: PLoS One. 2024 Feb 27;19(2):e0298648. doi: 10.1371/journal.pone.0298648 (PMC10898745; doi:10.1371/journal.pone.0298648)
Supplement: S10 Table — Log-transformed RTs are set as the dependent variable. Language group (i.e., “Agrigentino”, “Pavese”, “bilingual”), “Judgement”, “% of use of Italian”, “% of use of the L2”, and “% of switching” are set as fixed factors in the model and their interactions with RTs are also reported. Animacy, register, gender, and age are set as control factors. (PDF) [file pone.0298648.s010.pdf]

Effect	Estimate	SE	t	p	By-participant SD	By-item SD
Intercept	3.1778239	0.03784051	83.97942	2.203706	0.1775	0.0323
Comparison between bilingual and Pavese groups in RTs	-0.135994	0.04955025	-2.74457*	0.007774		
Comparison between bilingual and Agrigentino groups in RTs	0.0693015	0.04489181	1.543744	0.127362		
Judgement	0.0426497	0.00653476	6.526599	7.855467		
% of language switching	-0.031112	0.02740176	-1.1354	0.26027		
% Italian language use	-0.014504	0.02689215	-0.53934	0.591441		
% L2 language use	-0.092019	0.04048825	-2.27273*	0.026263		
Animacy	-0.000352	0.00694745	-0.05071	0.959828		
Register	0.0014101	0.00695588	0.202717	0.840462		
Gender	0.0148799	0.02562078	0.580775	0.563345		
Age	0.0721191	0.02245913	3.211127*	0.002028		
Judgement * Comparison between bilingual and Pavese groups in RTs	0.0073624	0.00991194	0.742776	0.457678		
Judgement * Comparison between bilingual and Agrigentino groups in RTs	-0.008549	0.00835691	-1.02297	0.306402		
% of switching - Comparison between bilingual and Pavese groups in RTs	0.0955539	0.04098093	2.331667*	0.022743		
% of switching - Comparison between bilingual and Agrigentino groups in RTs	0.0236268	0.03682924	0.641523	0.523377		
% of use of Italian - Comparison between bilingual and Pavese groups in RTs	0.0221724	0.03934398	0.563552	0.574945		

% of use of Italian - Comparison between bilingual and Agrigentino groups in RTs	-0.054767	0.03488127	-1.57009	0.1211
% of use of L2 - Comparison between bilingual and Pavese groups in RTs	-0.095244	0.0621837	-1.53166	0.130326
% of use of L2 - Comparison between bilingual and Agrigentino groups in RTs	0.0121473	0.05594454	0.217131	0.828767

S10 Table. Fixed and random effects from the second LME of RTs, with the bilingual group as the baseline. Log-transformed RTs are set as the dependent variable. Language group (i.e., “Agrigentino”, “Pavese”, “bilingual”), “Judgement”, “% of use of Italian”, “% of use of the L2”, and “% of switching” are set as fixed factors in the model and their interactions with RTs are also reported. Animacy, register, gender, and age are set as control factors.
