# Supplementary material for: The role of minority language bilingualism in spotting agreement attraction errors: Evidence from Italian varieties
Source: PLoS One. 2024 Feb 27;19(2):e0298648. doi: 10.1371/journal.pone.0298648 (PMC10898745; doi:10.1371/journal.pone.0298648)
Supplement: S11 Table — Log-transformed RTs are set as the dependent variable. Language group (i.e., “Pavese”, “bilingual”, “Agrigentino”), “Judgement”, “% of use of Italian”, “% of use of the L2”, and “% of switching” are set as fixed factors in the model and their interactions with RTs are also reported. Animacy, register, gender, and age are set as control factors. (PDF) [file pone.0298648.s011.pdf]

Effect	Estimate	SE	t	p	By-participant SD	By-item SD
Intercept	3.177824	0.037841	83.97942	2.203701	0.1775	0.0323
Comparison between Agrigentino and Pavese groups in RTs	-0.13599	0.04955	-2.74457*	0.007774		
Comparison between bilingual and Agrigentino groups in RTs	0.066693	0.066288	1.006112	0.317993		
Judgement	0.04265	0.006535	6.526599	7.855467		
% of language switching	-0.03111	0.027402	-1.1354	0.26027		
% Italian language use	-0.0145	0.026892	-0.53934	0.591441		
% L2 language use	-0.09202	0.040488	-2.27273*	0.026263		
Animacy	-0.00035	0.006947	-0.05071	0.959828		
Register	0.00141	0.006956	0.202717	0.840462		
Gender	0.01488	0.025621	0.580775	0.563345		
Age	0.072119	0.022459	3.211127*	0.002028		
Judgement * Comparison between Agrigentino and Pavese groups in RTs	0.007362	0.009912	0.742776	0.457678		
Judgement * Comparison between bilingual and Agrigentino groups in RTs	0.001187	0.00893	0.132866	0.894308		
% of switching - Comparison between Agrigentino and Pavese groups in RTs	0.095554	0.040981	2.331667*	0.022743		
% of switching - Comparison between bilingual and Agrigentino groups in RTs	-0.11918	0.041492	-2.87239*	0.005457		
% of use of Italian - Comparison between Agrigentino and Pavese groups in RTs	0.022172	0.039344	0.563552	0.574945		

% of use of Italian - Comparison between bilingual and Agrigentino groups in RTs	0.032594	0.038082	0.85589	0.395095
% of use of L2 - Comparison between Agrigentino and Pavese groups in RTs	-0.09524	0.062184	-1.53166	0.130326
% of use of L2 - Comparison between bilingual and Agrigentino groups in RTs	0.083097	0.054365	1.528498	0.13111

S11 Table. Fixed and random effects from the second LME of RTs, with the Italian-Agrigentino bidialectal group as the baseline. Log-transformed RTs are set as the dependent variable. Language group (i.e., “Pavese”, “bilingual”, “Agrigentino”), “Judgement”, “% of use of Italian”, “% of use of the L2”, and “% of switching” are set as fixed factors in the model and their interactions with RTs are also reported. Animacy, register, gender, and age are set as control factors.
