# Supplementary material for: The role of minority language bilingualism in spotting agreement attraction errors: Evidence from Italian varieties
Source: PLoS One. 2024 Feb 27;19(2):e0298648. doi: 10.1371/journal.pone.0298648 (PMC10898745; doi:10.1371/journal.pone.0298648)
Supplement: Attachment — Submitted filename: Response to Reviewers.docx [file pone.0298648.s013.docx]

**Response letter**

We would like to thank the Editor, Prof. Montserrat Comesaña Vila, and all the Reviewers for their positive feedback on our revised work. In this letter, we address all the points raised by the second Reviewer. The changes made to address the Reviewer’s observations appear in red in the revised version of the manuscript and are also reported in this response letter.

**Comments by Reviewer 2**

*I appreciate the authors’ care in addressing the many comments that I submitted. While I think that the manuscript has improved substantially, I think some aspects of the study remain to be clarified. They are outlined below.*

- *Participants*

*In their revision and response, the authors did not clarify why 152/278 participants did not complete the task. I think this information should be included in the manuscript.*

We added the required clarification on page 10 of the revised version of the manuscript.

P. 10: “In particular, the 152 participants who did not complete the task were excluded because they only filled one section of the experiment, namely the background questionnaire, without starting or, in some cases, completing the acceptability judgement task before the end of the data collection session.”

- *Stimuli*

*Did the authors create the stimuli? Or where borrowed from previously published work? Please clarify and cite pertinent work, if needed.*

This piece of information has been added to the revised version of the manuscript, on page 12.

P. 12: “The stimuli were specifically created for this study and constitute original material available at: https://osf.io/j4zqg/?view_only=e52f1e4facb9474984148cefac087b51.”

- *Variables*
- *In their response, the authors wrote that the DV concerned their performance to the critical 40 ungrammatical sentences, in terms of accuracy and RT. They also wrote “Data analyses included both accurate and inaccurate responses to the test items” (p. 15, lines 341-342) and “Moreover, we examined whether the acceptability rates influence the RTs” (p. 17, line 382). I am not sure how acceptability rates can influence RT, as we are discussing two dependent variables and a relationship about covariation.*

Although acceptability rates constitute the dependent variable of one set of our analyses (“Accuracy analyses”, pp. 16, 18, 21), they are not set as dependent variables in all the models included in our analyses. In the analyses of RTs, where RTs were set as the dependent variable (“RT analyses”, pp. 17, 19, 22), we decided to include acceptability rates as a fixed factor, as specified on page 17 (“As fixed effects, we included…the acceptability judgement given to the stimulus (scaled)”), to observe any potential relation between distinct judgements on a 5-point Likert scale and the corresponding RTs. Thus, in the case of RT analyses, acceptability rates are not the dependent variable anymore. They are instead treated as a potential factor modulating RTs. To clarify this point, we changed the phrasing of the sentence cited by the Reviewer as follows:

P. 17: “Moreover, we examined whether ~~the acceptability rates influence the~~ RTs were modulated by acceptability judgements.”

- *Also, it is still unclear to me why RT analyses were calculated for both accurate and inaccurate responses. Given that the main hypotheses in the study concern attraction errors, the analyses could have focused on the RT of accurate responses (answers 1 and 2 to sentences with attraction errors). Why including RT for answers 3, 4, 5? What is the justification, theoretically speaking? Much of the Results and Discussion sections concerns differences in RT between groups, so I think it is very important to explain this choice.*

We have added a paragraph on page 15 of the revised manuscript, including two new references [86, 87], to address this comment from the Reviewer.

P. 15: “The inclusion of both accurate and inaccurate responses and their corresponding RTs was done to comprehensively observe participants’ behavior regarding agreement attraction errors, which is the main purpose of this study. By including RTs of both accurate and inaccurate responses, we seek to highlight potential trends in the time needed to give (in)correct responses, which have been highlighted in previous literature [60, 86]. In particular, recent research on the processing of Subject-Verb agreement mismatches showed that inaccurate judgements are associated with slower RTs compared to accurate judgements [87]. Furthermore, given that Italian-Pavese and Italian-Agrigentino bidialectals have never been examined in the processing of Subject-Verb agreement mismatches, and in general, in language processing research, we opted not to exclude a priori a significant portion of our database to entirely observe the processing behavior of these unstudied populations”.

- *Analyses*
- *P. 16: Information about how the authors dealt with convergence issues should be added to the paper. (“To be more specific, we started by removing the interactions in the slopes, then we proceeded to remove the slopes with lower explained variance until convergence was reached.”)*

We added this piece of information in the revised version of the manuscript.

P. 16: “To be more specific, we started by removing the interactions in the slopes, then we proceeded to remove the slopes with lower explained variance until convergence was reached”.

P. 17: “Again, we started by removing the interactions in the slopes, then we removed the slopes with lower explained variance until reaching convergence”.

- *Pp. 21-22: RTs analyses. I assume that both accurate and inaccurate trials were included? [please see my comment in the “Variables” section.]*

Yes, both accurate and inaccurate trials were included. As we specified in the answer to the Reviewer’s comment in the “Variables” section of the present Response Letter, we have added a paragraph on page 15 of the revised version of the manuscript. In this new paragraph, we explain our choice of including RTs from both accurate and inaccurate acceptability judgements.

- *Figures*
- *All figures should include information on how variability in the data is represented (e.g., SD, SE). They only include the variables is on the X and Y axes.*

The required pieces of information have been added to all captions of the revised version of the manuscript.

P. 18: “**Fig 1. Accuracy rates for each language group.** The x-axis represents the language groups, while the y-axis shows the mean accuracy rates from 0 (i.e., “Inaccurate”) to 1 (i.e., “Accurate”). The vertical lines represent standard errors”.

P. 20: “**Fig 2. RTs for each language group.** The x-axis represents the language groups, while the y-axis shows RTs in milliseconds recorded for each language group. The vertical lines represent standard errors”.

P. 20: “**Fig 3. Distribution of RTs for each language group.** The x-axis represents ~~how~~ the distribution of ~~judgements aligns with~~ RTs across various language groups. The y-axis reports RTs in milliseconds. The violin shapes represent data density, while the box plots represent standard deviations”.

P. 21: “**Fig 4. ~~Interaction between RTs and Judgement for each language group~~.** **Interaction between Acceptability Judgment and Language group on RTs.** The x-axis shows the acceptability judgements given to the stimuli. The y-axis reports RTs in milliseconds. The error ribbons represent 95% confidence intervals”.

P. 23: “**Fig 5. Main effect of the percentage of L2 use on RTs ~~Interaction between the percentage of L2 use and language group~~.** The x-axis shows the percentage of time using the L2, while the y-axis reports RTs in milliseconds. The error ribbon represents 95% confidence interval”.

P. 23: “**Fig 6. Interaction between the percentage of language switching and Language group.** The x-axis shows the percentage of language switching. The y-axis reports RTs in milliseconds. The error ribbon represents 95% confidence interval”.

- *P. 19, Figure 3. The legend states the following: “Fig 3. Distribution of RTs for each language group. The x-axis represents how the distribution of judgements aligns with RTs across various language groups. The y-axis reports RTs in milliseconds.” I am not sure I understand what alignment means here, as the various types of judgments are not clearly distinguishable.*

Thanks to this Reviewer’s comment, we modified the caption of Figure 3 as follows:

P. 20: “**Fig 3. Distribution of RTs for each language group.** The x-axis represents ~~how~~ the distribution of ~~judgements aligns with~~ RTs across various language groups. The y-axis reports RTs in milliseconds. The violin shapes represent data density, while the box plots represent standard deviations.”.

- *P. 20, Figure 4. I commented on the relationship between these two dependent variables in the Variables section. Regardless, I wonder if instead of “Fig 4. Interaction between RTs and Judgement for each language group” are more apt title would be ““Fig 4. Relationship between RTs and Judgement for each language group.”*

We appreciate the Reviewer’s suggestion. Still, we believe it is important to remark that the figure represents the significant interaction between the acceptability judgments and language groups found in the statistical model of RTs. We modified the caption as follows:

P. 21: “**Fig 4. ~~Interaction between RTs and Judgement for each language group~~.** **Interaction between Acceptability Judgment and Language group on RTs.** The x-axis shows the acceptability judgements given to the stimuli. The y-axis reports RTs in milliseconds. The error ribbons represent 95% confidence intervals.

- *P. 22, Figure 5. “Fig 5. Interaction between the percentage of L2 use and language group. The x-axis shows the percentage of time using the L2, while the y-axis reports RTs in milliseconds.” There is only one factor represented in the figure (percentage of language used).*

We changed the caption of Figure 5 as follows:

P. 23: “**Fig 5. Main effect of the percentage of L2 use on RTs ~~Interaction between the percentage of L2 use and language group~~.** The x-axis shows the percentage of time using the L2, while the y-axis reports RTs in milliseconds. The error ribbon represents 95% confidence interval.”.

- *Discussion*
- *P. 29: “In particular, we find a positive correlation between RTs and the percentage of L2 use.” The correlation is negative, based on Fig. 5: the higher the L2 use, the shorter the RT. Also, based on previous analyses, I assume that RTs were calculated on all five types of responses, thus including inaccurate responses. If this is incorrect (so, only accurate responses were included), please disregard this comment. If it is correct, again, I wonder what the inclusion of both correct and incorrect answers tells us, in terms of the hypotheses assessed here.*

Thanks to the Reviewer’s comment, we substituted the adjective “positive” with “negative” in the revised version of the manuscript.

P. 30: “In particular, we find a negative ~~positive~~ ~~correlation~~ relation between RTs and the percentage of L2 use.”.

As commented in earlier sections of this response letter, we opted to incorporate RTs for both accurate and inaccurate responses to ensure the inclusion of significant data portions in our analyses. Since Italian-Pavese and Italian-Agrigentino bidialectals have never been tested on an AJT involving Subject-Verb agreement mismatches, we wanted to observe behavioral results in their completeness, without precluding insights coming from incorrect responses. Furthermore, if we had excluded incorrect judgements (i.e., 3, 4, and 5), it would have been impossible to highlight the trend illustrated in Figure 4 (i.e., longer RTs for incorrect judgements).

In the specific case of the relation between RTs and L2 use, we considered the possibility of a clearer distinction between languages among participants who use their L2 more frequently. This distinction could result in reduced processing costs, attested by faster RTs, for evaluating a sentence presented exclusively in one of their linguistic systems, regardless of the accuracy of the judgment. Including both correct and incorrect responses allowed us to observe the general RT tendencies of different bilingual and bidialectal groups concerning specific language use variables.

- *I am unclear about the last paragraph on p. 29. The discussion concerns the data in Fig. 6, with two different patterns of RTs in Pavese (positive correlation with percentage of switching) and standard bilinguals (negative correlation). I am not sure what the following sentence refers to: “However, the absence of significant correlations between RTs and accuracy does not allow further speculations on the possible advantages of language switching for our bilingual participants.” Please clarify.*

The sentence has been modified in the revised version of the manuscript.

Pp. 30-31: “However, the absence of a main effect of switching and the lack of interaction between switching, the opposite pattern found in Italian-Spanish bilinguals and Italian-Pavese bidialectals, and language group in the further analysis of bilinguals’ and bidialectals’ accuracy rates do~~es~~ not allow further speculations on the possible advantages of language switching for our bilingual participants.”

- *Finally, in their response, the authors wrote:“We apologise for the confusion created by the use of inaccurate terminology in the previous version of the manuscript. This is not a correlation study. For the analyses, we used regression models, which reveal whether and in what way the independent variables (i.e., fixed effects) have an impact on the dependent ones (accuracy of the judgments and response time). Differently from correlation analyses, which assess the strength of the relationship between two variables, regression analyses uncover causal relationships between the variables, enabling us to discern the effects they exert on one another. Hence our choice to use terms such as “effect” and “impact”. Perhaps I am adopting a conservative approach, but if there is no manipulation (here, for example, language experience, gender, age are all subject variables), we cannot speak of causality. Causality is inferred based on the design of the study, not the statistical analyses used. So, I advise caution in both describing statistical effects and drawing conclusions when causality is drawn from a study with a non-experimental design.*

We thank the Reviewer for giving us the possibility of clarifying this point. As the Reviewer correctly pointed out, we should make clear that the relations between variables are not necessarily causal. In this light, we modified some parts of the Result and Discussion sections, to soften any statement about the interaction between variables that could have been interpreted as a strong causal relation. In this way, we delineate that the variation of specific factors within the bilingual experience can coincide with changes in accuracy/response times (RTs), without necessarily suggesting causal relationships.

Pp. 20-21: “Interestingly, there is an interaction between acceptability judgements and language groups (t = -2.25, S5 Table, Supporting Information), revealing that while ~~a significant difference between the Italian-Agrigentino bidialectal group is slower and than the monolingual group (t = -2.25, S5 Table, Supporting Information)~~. ~~As Fig 4 shows,~~ monolingual participants show a prominent difference between RTs associated with accurate vs. inaccurate judgements, such that inaccurate judgements are associated with longer RTs, the difference is less pronounced for Italian-Agrigentino bidialectal speakers.”

P. 22: “Our second GLME with accuracy as the dependent variable was run to determine whether the percentage of use of Italian vs. the other majority or minority language, together with the frequency of language switching, modulates ~~has an impact on~~ accuracy rates.”

P. 22: “we are interested in seeing whether variables related to the language practices of bilingual participants with minority vs. majority languages ~~have an impact on~~ modulate RTs.”

P. 30: “our findings demonstrate that language processing outcomes can ~~be attributed to~~ significantly change together with variables associated with specific language practices.”

P. 30: “In particular, we find a negative ~~positive~~ ~~correlation~~ relation between RTs and the percentage of L2 use.”

P. 30: “Besides the percentage of L2 use, another sociolinguistic factor that seems to play~~s~~ a role on RTs is language switching.”

P. 30: “The ~~positive correlation~~ negative relation between switching and response latencies may suggest ~~support the hypothesis~~ that the constant juggling between two languages trains the parser.”
